# *piggyBac* Transposition and the Expression of Human Cystatin C in Transgenic Chickens

**DOI:** 10.3390/ani11061554

**Published:** 2021-05-26

**Authors:** Seo Woo Kim, Jeong Hyo Lee, Ji Seon Han, Seung Pyo Shin, Tae Sub Park

**Affiliations:** 1 Graduate School of International Agricultural Technology, Seoul National University, Pyeongchang-gun 25354, Gangwon-do, Korea; ektmfrl0125@snu.ac.kr (S.W.K.); hanjiseon@snu.ac.kr (J.S.H.); 2 Institute of Green-Bio Science and Technology, Seoul National University, Pyeongchang-gun 25354, Gangwon-do, Korea; megimetel@snu.ac.kr (J.H.L.); sinseng1234@snu.ac.kr (S.P.S.)

**Keywords:** *piggyBac* transposon, human cystatin C, transgenic chickens, bioreactor

## Abstract

**Simple Summary:**

The genetic modification of livestock genomes showed the great potential for production of industrial biomaterials as well as improving animal production. Particularly, the transgenic hen’s eggs have been considered for a massive production system of the genetically engineered biomaterials as a bioreactor animal. Virus-mediated transgene transduction is the most powerful strategy to generate the transgenic animals. However, industrial applications were hampered by many obstacles such as relatively low germline transmission and transgene silencing effects, as well as viral safety issues. In this study, a *piggyBac* transposon which is a non-viral integration technical platform was introduced into chicken primordial germ cells. Finally, we developed transgenic chickens and assayed the bioactivity of human cystatin C in the transgenic chicken’s tissues.

**Abstract:**

A bioreactor can be used for mass production of therapeutic proteins and other bioactive substances. Although various methods have been developed using microorganisms and animal cells, advanced strategies are needed for the efficient production of biofunctional proteins. In microorganisms, post-translational glycosylation and modification are not performed properly, while animal cell systems require more time and expense. To overcome these problems, new methods using products from transgenic animals have been considered, such as genetically modified cow’s milk and hen’s eggs. In this study, based on a non-viral *piggyBac* transposition system, we generated transgenic bioreactor chickens that produced human cystatin C (hCST3). There were no differences in the phenotype or histochemical structure of the wild-type and hCST3-expressing transgenic chickens. Subsequently, we analyzed the hCST3 expression in transgenic chickens, mainly in muscle and egg white, which could be major deposition warehouses for hCST3 protein. In both muscle and egg white, we detected high hCST3 expression by ELISA and Western blotting. hCST3 proteins were efficiently purified from muscle and egg white of transgenic chickens using a His-tag purification system. These data show that transgenic chickens can be efficiently used as a bioreactor for the mass production of bioactive materials.

## 1. Introduction

A bioreactor is a device or system that supports a biologically active environment for the mass production of biofunctional proteins. Bioreactors include prokaryotic and eukaryotic cell systems and transgenic animals [1,2]. Using recombinant DNA (rDNA) technology, recombinant human insulin produced in *E*. *coli* was approved by the US Food and Drug Administration (FDA) for clinical use and was the first commercial recombinant pharmaceutical protein [2]. However, due to inaccurate post-translational modification in bacteria and yeast bioreactor systems, recombinant pharmaceutical proteins have reduced efficacy and limited shelf life, and cause undesired side effects [3,4,5,6]. Thus, animal cell systems are used to produce recombinant therapeutic proteins, but the production levels are relatively low and costly compared to other systems [3,4,5,6].

Transgenic bioreactor animals might be an alternative avenue through which therapeutic proteins could be produced. The application of transgenic animal technology could be extended to produce desirable proteins stably via in vivo expression systems [3,4,5,6,7,8,9,10,11]. In 2009, the FDA first approved recombinant human anti-thrombin-α protein produced from milk of transgenic goats for human treatment [12]. The genetically engineered animals created considerable excitement for industrial application for producing medicines. Particularly, transgenic chickens as a bioreactor system have advantages such as a short generation time, easy pedigree maintenance, and large production capacity [7,8,9,10,11]. Germ cell-mediated transgene transfer can be used to generate transgenic chickens because chicken primordial germ cells (PGCs) migrate through blood vessels and localize to the developing genital ridges during early embryonic stages [13,14,15,16]. Combined with a chicken PGC culture system, efficient non-viral transgene delivery with *piggyBac* and Tol2 transposon elements has been developed for the production of transgenic chickens [13,17].

Cystatin C, a member of the cystatin super family, is a 13 kDa polypeptide containing 120 amino acids [18,19,20]. It is produced in the body at a constant rate and is found in body fluids and secretions [18]. It inhibits cysteine protease and protects against microbial and parasitic infiltration [21,22,23]. For recombinant protein production in vitro, human cystatin C (hCST3) is produced mainly in eukaryotic cells such as HEK293 cell lines but the cell-based production system of biomaterials is too laborious and relatively expensive [1,2]. *E. coli* system could be utilized to produce hCST3 but the bacterial platform should be necessary of the extra modification step for formation of two disulfide bonds in hCST3 protein. However, there is no report on animal bioreactor for hCST3 production. In this study, using a non-viral *piggyBac* transposon system, we produced transgenic bioreactor chickens that express hCST3. The transgenic chickens expressed hCST3 throughout the body.

## 2. Materials and Methods

### 2.1. Experimental Animal Care

The procedures followed in the care of chickens for experimental use in this study were approved by the Institutional Animal Care and Use Committee (SNU-150825-2-1), Seoul National University. Chickens were maintained according to a standard management program at the University Animal Farm, Pyeongchang, Seoul National University, Korea. The procedures for animal management, reproduction, and embryo manipulation adhered to the standard operating protocols of our laboratory.

### 2.2. Codon Optimization of Human Cystatin C Gene and Construction of Expression Vector into piggyBac Transposon

Codon of human cystatin C (hCST3) gene was optimized based on *Gallus gallus* codon usage database (http://www.kazusa.or.jp/codon, accessed on 5 January 2016) and synthesized by Bionics Co. (Seoul, Korea). To insert hCST3 gene into *piggyBac* transposon transgene expression system vector (System Biosciences, Palo Alto, CA, USA), the synthesized hCTS3 gene was digested by *Nhe* I and *Not* I and subsequently ligated into *piggyBac* transposon.

### 2.3. Chicken DF1 Cell and PGC Culture for Transfection and Selection

Chicken DF1 cell line was preliminarily utilized to examine the expression of hCST3 protein. DF1 is a chicken embryonic fibroblast cell line that was spontaneously transformed and immortalized from 10-day-old embryo. A PGC line (Pw66) derived from 6-day-old male embryonic gonads of White Leghorn (WL) chickens was maintained and subpassaged according to our previous report [13,14]. Briefly, chicken PGCs were maintained with knockout Dulbecco’s modified Eagle’s medium (Invitrogen, Carlsbad, CA, USA) supplemented with 20% fetal bovine serum (FBS, Invitrogen, Carlsbad, CA, USA), 2% chicken serum (Sigma-Aldrich, St. Louis, MO, USA), 1x nucleosides (Millipore, Temecula, CA, USA), 2 mM L-glutamine, 1× nonessential amino acids, β-mercaptoethanol, 10 mM sodium pyruvate, and 1× antibiotic–antimycotic (Invitrogen, Carlsbad, CA, USA). Human basic fibroblast growth factor (bFGF; 10 ng/mL; Koma Biotech, Seoul, Korea) was used for PGC proliferation. Chicken PGCs were cultured in an incubator at 37 °C with an atmosphere of 5% CO_2_ and 60–70% relative humidity. The cultured PGCs were subcultured onto mitomycin-inactivated mouse embryonic fibroblasts (MEFs) in 5- to 6-day intervals by gentle pipetting without any enzyme treatment. The transfection procedure was conducted according to our previous report [15,16]. hCST3 and transposase expression vector were co-transfected into chicken PGCs or DF1 cells by lipofection with Lipofectamine 3000^®^ reagent (Invitrogen, Carlsbad, CA, USA). One day after transfection, 10 μg/mL puromycin was added to the culture media for selection. Additionally, to isolate single-cell-derived hCST3-expressing PGC sublines (hCST3-PGC#1-#5), a single PGC was picked up using a micropipette under the microscope (Nickon, Tokyo, Japan) and dropped onto a 96-well culture plate with an MEF feeder layer (CEFO Bio, Seoul, Korea). To examine the migration capacity in vivo, *piggyBac* GFP expression vector was also integrated into hCST3-PGC#3.

### 2.4. Transplantation of hCST3-Expressing Chicken PGCs and Testcross Analysis to Screen Transgenic Chicks

hCST3-PGC#3 without GFP transgene was transplanted into recipient embryos (Hy-Line Brown) because hCST3-PGC#3 showed higher expression of hCST3 by ELISA assay. For transplantation, a small window was made on the pointed ends of recipient eggs, and 3000 transgenic PGCs was microinjected into the dorsal aorta of each recipient embryo using a micropipette. The egg window of the recipient embryo was sealed with paraffin film, and the egg was incubated with the pointed end down until hatching. After sexual maturation, germline chimeras were identified through testcross analysis by mating with WL chickens. The offspring phenotype from endogenous germ cells in Hy-line Brown recipients showed hybrid chicks with brown or black spots, whereas WL donor hCST3-expressing germ cells produced only white chicks. Subsequently, transgenic chicks were screened and identified by genomic PCR. 

### 2.5. Detection of hCST3-Expressing Transgenic Chickens

To detect hCST3 transgene, two primer sets were designed and used for genomic PCR reaction (hCST3 F1 5′-CAC TGC TCC TCC TTG CGA TC-3′, hCST3 R1 5′-GGT GAT GGT GAT GGG CAT CC-3′, hCST3 F2 5′-GTG AAC CGT CAG ATC GCC TG-3′, hCST3 R2 5′-GTG AGG CTG GTC GTG GAA AG-3′). The PCR product size was 429 bp and 418 bp by hCST3 F1-R1 and hCST3 F2-R2, respectively. PCR reactions were performed with an initial incubation at 94 °C for 5 min, followed by 30 cycles at 94 °C for 30 s, 63 °C for 30 s and 72 °C for 30 s. The reactions were terminated by a final incubation at 72 °C for 5 min and PCR amplicons were identified by agarose gel electrophoresis.

### 2.6. ELISA for Detection of hCST3 Protein

hCST3 protein in cells and transgenic chickens was detected and the concentration was determined by using human cystatin C Quantikine^®^ ELISA Kit (R&D Systems, Minneapolis, MN, USA) according to the manufacturer’s protocol. 50 μL supernatant of DF1 or PGC culture were added to each microplate well containing 100 μL of assay diluent buffer. After incubation for 3 h at 4 °C, each well was aspirated and washed by 400 μL of wash buffer 4 times. Subsequently, each well was added 200 μL of cold human cystatin C conjugate and incubated for 1 h at 4 °C. After washing, 200 μL of substrate solution was added into each well and incubated for 30 min at room temperature in dark place. Finally, each well was added with 50 μL of stop solution and measured by ELISA reader. For in vitro assay of hCST3 in transgenic hen’s eggs, the egg white was diluted 1:100 with PBS and 50 μL of each sample was used for ELISA assay according to the same protocol for cell supernatant.

### 2.7. Western Blotting

Total proteins from feather pulp and homogenized muscle (*pectoralis major*) from wild-type and hCST3 transgenic chickens were extracted with 1× radioimmunoprecipitation (RIPA) lysis buffer (Rockland, Limerick, PA, USA). These samples separated on a 15% polyacrylamide gel followed by transfer to a nitrocellulose membrane. Additionally, we analysed hCST3 proteins after purification from muscle and egg white. The primary antibodies used were mouse anti-β-actin (Santa Cruz Biotechnology, Santa Cruz, CA, USA) or anti-human cystatin C (Abcam, Cambridge, UK). HRP-conjugated anti-mouse IgG or anti-rabbit IgG (Bio-Rad, Hercules, CA, USA) were used as secondary antibodies. The blots were treated with ECL substrate solutions and exposed in a ChemiDoc XRS System (Bio-Rad, Hercules, CA, USA) to detect chemiluminescence.

### 2.8. Immunohistochemistry Analysis

Muscles (pectoralis major), liver, and kidney of wild-type and hCST3 transgenic chickens were cut into small pieces (0.5 × 0.5 × 1.0 cm) and fixed in formalin [10% (*v*/*v*) formaldehyde in phosphate-buffered saline] for histological examination. Paraffin-embedded tissue blocks were obtained, and sections (4 µm thick) from the mid-portions of wounds were stained with hematoxylin-eosin (H&E, Vector Labs, Burlingame, CA, USA) after deparaffinization. All slides were histologically observed under an Aperio AT2 slide scanner (Leica Biosystems, Wetzlar, Germany). 

### 2.9. Purification of hCST3 from Egg White and Muscle

Transgenic hen’s egg white or homogenized muscle (pectoralis major) was diluted 1:100 with PBS for purification using MagListo™ His-tagged protein purification kit (Bioneer Co. Daejeon, Korea). 500 μL of each sample was mixed with the pre-equilibrated Ni-NTA magnetic silica resin and placed on a Nd magnet for 1 min. After washing with binding/washing buffer, 100 μL of elution buffer was added to collect the purified hCST3 eluate.

### 2.10. Antimicrobial Activity Test

Antimicrobial activities were determined by a disk diffusion method according to Wesierska et al. [24]. The purified hCST3 from transgenic hen’s egg white was transferred in Whatman paper disks at different concentrations (10, 25, 50, 75, 100 ng/disk). *E. coli* was streaked in four directions with a swab on LB agar plate and hCST3 disks were applied to the bacterial-seeded plates. Each plate was incubated at 37 °C for 24 h and the inhibition zones were observed to determine whether the purified hCST3 possessed bactericidal activity.

## 3. Results

### 3.1. Codon-Optimization and Construction of hCST3 Expression Vector in piggyBac Transposon

**Figure 1:**
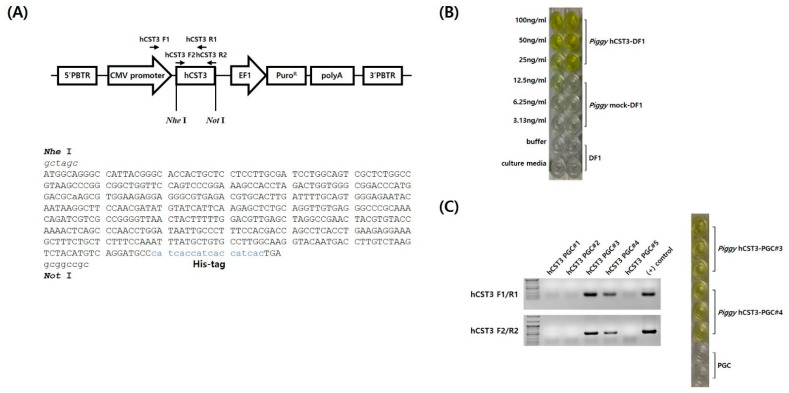
The *piggyBac* transposon-based vector and expression of human cystatin C (hCST3). (**A**) Schematic of the expression vector and chicken codon-optimized hCST3 with a His-tag. The CMV promoter controlled hCST3 expression and a puromycin-resistance gene was used as a transgene selection marker. The arrows indicate the primer sites in the hCST3 expression vector (hCST3, human cystatin C; PBTR, *piggyBac* terminal repeat; CMV, cytomegalovirus; EF1, elongation factor 1). (**B**) Detection of hCST3 expression in transfected, puromycin-selected DF1 cell lines. (**C**) Detecting the hCST3 transgene and its expression in transfected, puromycin-selected chicken primordial germ cells (PGCs).

The hCST3 nucleotide sequences were codon-optimized for chicken cell expression and synthesized to construct a *piggyBac* transposon-based expression vector (Figure 1A). For hCST3 secretion from cells, chicken lysozyme signal peptide sequences were also synthesized upstream from the hCST3 gene. A His-tag (His6) was added in front of the hCST3 stop codon for efficient purification. hCST3 expression was regulated by the cytomegalovirus (CMV) promoter and the elongation factor 1 (EF1) promoter was used to express a puromycin-resistance gene for the stable selection of hCTS3 (Figure 1A).

### 3.2. Detection of hCST3 in DF1 Cells and PGCs after Transfection and Selection

To examine the expression of chicken codon-optimized hCST3, an expression vector with a ubiquitous CMV promoter was transfected into DF1 cells and selected with puromycin for up to 2 weeks. hCST3 expression was detected in the DF1 cell culture medium by ELISA, indicating that the codon-optimized hCST3 was expressed constantly and secreted from chicken cells (Figure 1B). Subsequently, we established five PGC-derived sublines using single-cell pick-up and in vitro expansion. Based on genomic PCR analyses of the five single PGC-derived sublines, we ultimately selected two hCST3-expressing PGC lines in which hCST3 expression was detected by ELISA (hCST3-PGC#3 and hCST3-PGC#4, Figure 1C).

### 3.3. Production of Transgenic Chickens by Transplanting hCST3-Expressing Chicken PGCs

**Figure 2:**
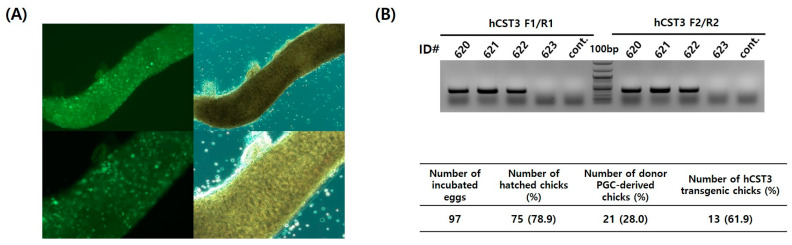
Production of hCST3 transgenic chickens. (**A**) Detection of GFP-expressing hCST3-transgenic chicken PGCs. GFP-positive chicken PGCs were detected in the recipient embryonic gonads (6-day-old) after transplantation. (**B**) Production of hCST3 transgenic chickens through testcross analyses. Ultimately, 13 hatched chicks were identified as transgenic chickens by genomic DNA PCR analyses. ID# is the individual number of donor PGC-derived chicks.

To examine the migration capacity of hCST3-PGC#3, the GFP transgene was also integrated into hCST3-PGC#3 and transferred into recipient embryonic blood vessels. After transplantation, GFP-expressing PGCs were detected in recipient gonads of 6-day-old embryos (Figure 2A). Finally, through testcross analyses, transgenic chicks with the *piggyBac* hCST3 transgene were identified from donor germ cell (hCST3-PGC#3)-derived offspring by genomic DNA PCR analyses (Figure 2B). A germline chimeric male chicken showed 28.0% (21/75) donor germ cell-derived germline transmission (Figure 2B); of the 21 (61.9%) donor germ cell-derived chicks (F_1_), 13 were identified as hCST3 transgenic chicks (Figure 2B).

### 3.4. Immunohistochemistry Analyses of hCST3-Expressing Transgenic Chickens

**Figure 3:**
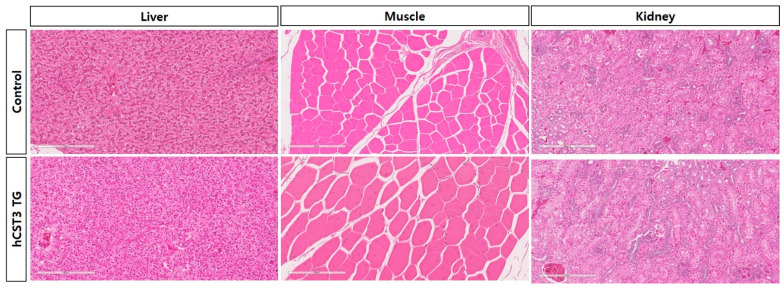
Comparative histochemistry analyses of hCST3 transgenic chickens. There were no differences in the histochemical structure of the liver, muscle, or kidney between control and hCST3 transgenic chickens. Paraffin sections were stained with hematoxylin and eosin (magnification 20×).

There were no significant differences in phenotype or growth performance between the wild-type (WT) and hCST3 transgenic chickens. Next, we conducted immunohistochemical analyses of the hCST3 transgenic chickens to look for abnormalities and structural dysfunction. Three major organs (muscle, liver, and kidney) were subject to paraffin sectioning and immunohistochemical assays. We did not observe any phenotypic difference in histochemical structure in any organ tissue between WT and hCST3 transgenic chickens (Figure 3).

### 3.5. Detection of hCST3 in Transgenic Chickens

**Figure 4:**
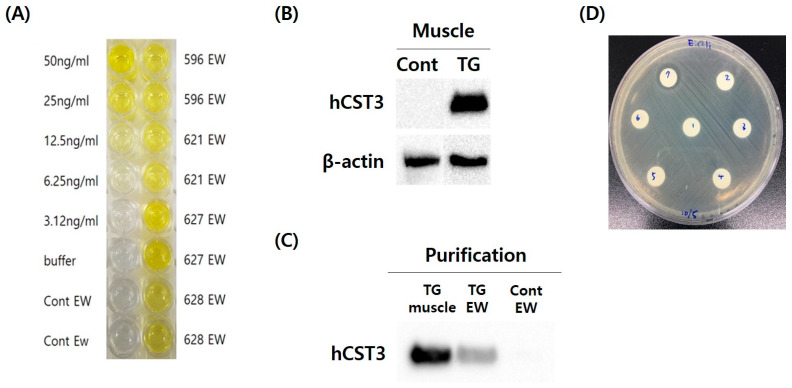
Quantification and antimicrobial activity test of hCST3 in transgenic chickens. (**A**) Quantification of hCST3 in the egg white (EW) of transgenic hens by ELISA. hCST3 was detected in all of the transgenic hen eggs. (**B**) hCST3 was detected in the muscle of hCST3 transgenic chickens by Western blotting. (**C**) His-tagged hCST3 in muscle and egg white of hCST3 transgenic chickens was purified and detected by Western blotting after purification with Ni-NTA magnetic nanobeads. (**D**) Biofunctional activity of hCST3 from transgenic hen eggs. The purified hCST3 from transgenic hen egg white was transferred to Whatman paper disks at different concentrations: (1) control (ddH_2_O), (2) elution buffer, and (3) 10, (4) 25, (5) 50, (6) 75, and (7) 100 ng hCST3/disc. Original western blot figures in Figure S1.

An ELISA assay showed hCST3 protein expression in whole blood and feather pulp of the hatched hCST3 transgenic chicks (data no shown). After sexual maturation, we detected hCST3 protein in muscle and egg white by ELISA and Western blotting (Figure 4A,B). In hCST3 transgenic hen eggs, the expression ranged from 1.3 to 3.8 μg/mL indicating that all of the transgenic hens deposited hCST3 protein in their egg whites (Figure 4A). The hCST3 protein was also examined in *pectoralis major* muscle by Western blotting and significant expression was observed only in hCST3 transgenic chickens (Figure 4B). These results confirmed that the ubiquitous CMV promoter could be used to produce foreign proteins in transgenic chickens, including various organ tissues and egg white.

### 3.6. Purification of hCST3 and Antimicrobial Activity Test

hCST3 proteins from egg white and muscle of transgenic chickens were purified using a His-tag-based Ni-NTA magnetic bead purification system. Subsequently, we confirmed the purified hCST3 from egg white and muscle by Western blotting (Figure 4C). This showed hCST3 protein from both egg white and muscle, suggesting that a large quantity of foreign protein could be produced in these. To examine biofunctional activity, an antimicrobial activity test was used to measure the growth inhibition of *E*. *coli* after purification and dialysis of hCST3. *E*. *coli* growth was inhibited by 100 ng purified, dialyzed hCST3 from transgenic hen egg white (Figure 4D).

## 4. Discussion

The chicken is a valuable experimental model in developmental biology and pathology [25]. Recent advances in the CRISPR-Cas9 technical platform will facilitate the utilization of genetically modified chickens in industrial applications and as model animals [15,16]. Many reports on the generation of transgenic bioreactors in chickens have shown potential applications for the industrial production of large amounts of pharmaceutical proteins and biomaterials [7,8,9,10,11]. Chicken proteins lack α1–3-galactose epitopes [5,26,27]. Compared to the synthesis of therapeutic proteins in the mammary glands of transgenic goats and cattle, the glycosylation patterns of proteins in humans are different, indicating that α1–3-galactose epitopes are not found in tissues and secreted glycoproteins in humans [5,27]. Thus, the recombinant proteins produced in chickens could reduce the potential risk for adverse immune responses [5,27].

A transgenic chicken bioreactor is attractive because commercial hens lay eggs that contain approximately 6.5 g protein each almost every day [5,11]. For the mass deposition of foreign transgene products into egg white, the ovalbumin promoter, which is a major egg white protein (approximately 2 g total protein), was cloned and used for specific transgene expression in oviduct tubular gland cells of hens [5,6,7,8,9,10,11]. Transgenic chickens expressing a single chain Fv-Fc fusion protein deposited human immunoglobulin in egg white at levels ranging from 0.1 to 1.5 mg/mL [8]. Lillico et al. generated two transgenic lines expressing human interferon β1a (3.5 to 426 μg/mL) and a humanized single chain Fv-Fc mini-antibody (average 38 μg/mL) [9]. Recently, Herron et al. reported the efficient production of functional cytokines as a pharmaceutical protein [10]. In a previous study, we found that homozygous transgenic chickens deposited 20 ng/mL human epithelial growth factor in egg white [11].

The CRISPR-Cas9 system will advance the application of transgenic bioreactor chickens by integrating the targeted transgene into the specific ovalbumin gene locus. Combined with the CRISPR-Cas9 protocol, Oishi et al. successfully knocked-in the human interferon β gene into the chicken ovalbumin locus, resulting in the abundant expression of an exogenous protein in egg white [27]. As an alternative transgene expression system, we used the ubiquitous cytomegalovirus (CMV) promoter for the human cystatin C (hCST3) gene, instead of a tissue-specific promoter. Thus, the transgene was expressed in all body tissues of transgenic chickens and in egg white.

Cystatin C (CST3) is a kidney function biomarker [18,19]. The most commonly used kidney marker is creatinine, but some studies have suggested that CST3 is a better indicator of glomerular filtration than creatinine [19,20]. Acute kidney injury (AKI) in children and newborns is defined as a sudden decline in the glomerular filtration rate [20,28,29]. Thus, the early detection of AKI is crucial for appropriate treatment before the renal function declines significantly [20,28,29]. CST3 is a biomarker in AKI diagnostic protocols [20,28,29]. In this study, the kidney microstructure of hCST3-expressing chickens regulated by the ubiquitous CMV promoter did not show any abnormalities or dysfunction. In addition, CST3 can protect the body from viral and bacterial invasion, toxicity, and replication by inhibiting microorganism cysteine proteases [21,22,23]. We also found that hCST3 produced in transgenic chickens inhibited microorganism growth, suggesting that hCST3-expressing transgenic chickens could be applied in various fields to produce bioactive materials.

## 5. Conclusions

In this study, transgenic bioreactor chickens for hCST3 production were generated by a non-viral *piggyBac* transposon system, and hCST3 proteins were constantly expressed and stably deposited in muscle and egg white of transgenic chickens. This non-viral transgenic technical platform for poultry could be a promising and efficient approach for future practical applications in biopharmacy as well as agriculture industry.

## Data Availability

This study did not repart any data.

## Supplementary Materials

The following are available online at https://www.mdpi.com/article/10.3390/ani11061554/s1, Figure S1: Original western blot figures for Figure 4B,C.
