# Supplementary figures and images for: piggyBac Transposition and the Expression of Human Cystatin C in Transgenic Chickens
Source: Animals (Basel). 2021 May 26;11(6):1554. doi: 10.3390/ani11061554 (PMC8226945; doi:10.3390/ani11061554)

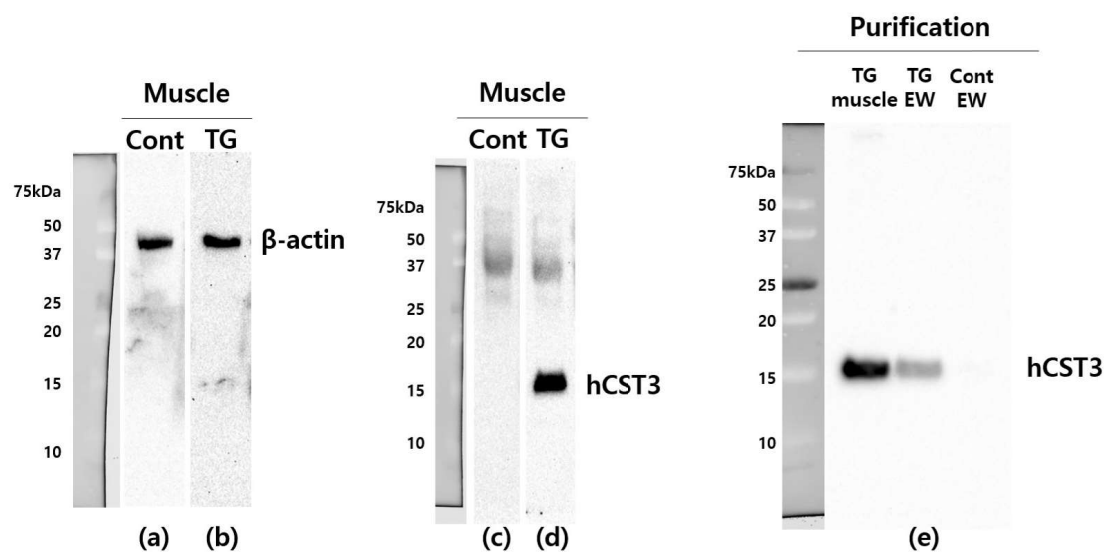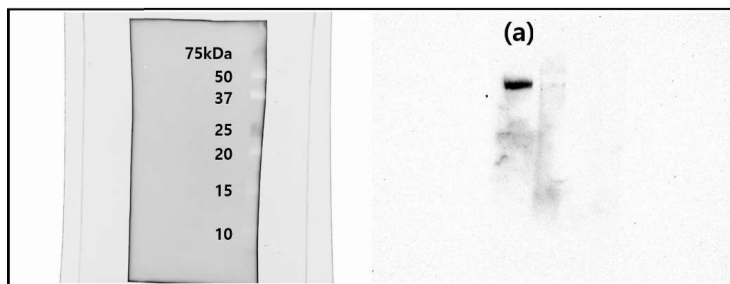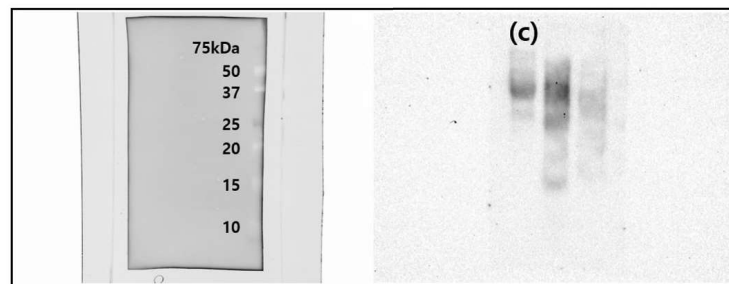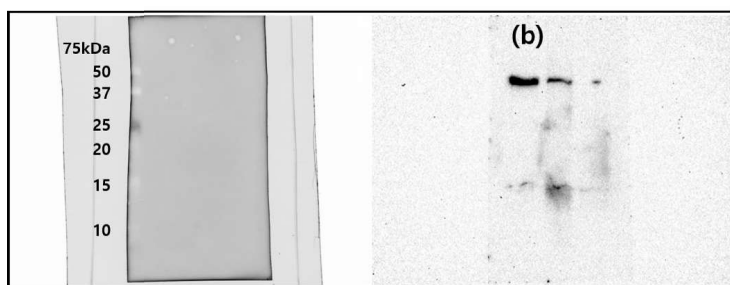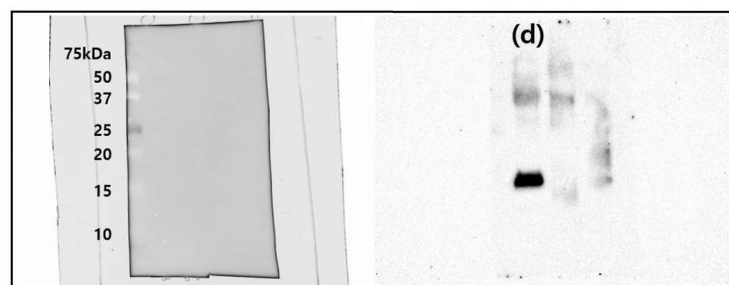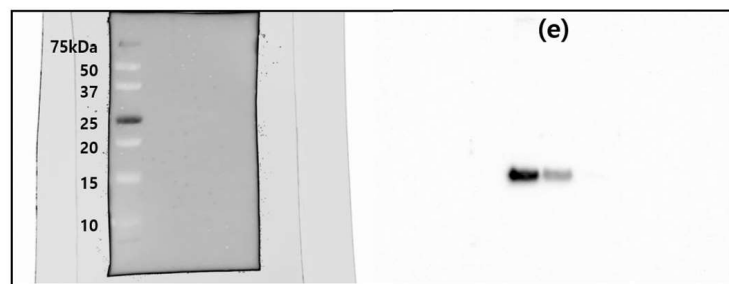

Supplement: Supplementary file 1 [file animals-11-01554-s001.zip › animals-1207987-supplementary.pdf]
